# Role of Laboratory Medicine in SARS-CoV-2 Diagnostics. Lessons Learned from a Pandemic

**DOI:** 10.3390/healthcare9070915

**Published:** 2021-07-19

**Authors:** Irena Duś-Ilnicka, Aleksander Szymczak, Małgorzata Małodobra-Mazur, Miron Tokarski

**Affiliations:** 1 Genomtec S.A., ul. Stabłowicka 147, 54-066 Wrocław, Poland; a.szymczak@genomtec.com (A.S.); m.malodobra-mazur@genomtec.com (M.M.-M.); m.tokarski@genomtec.com (M.T.); 2 Oral Pathology Department, Faculty of Dentistry, Wroclaw Medical University, ul. Krakowska 26, 50-425 Wrocław, Poland; 3 Hirszfeld Institute of Immunology and Experimental Therapy, Polish Academy of Sciences, ul. Rudolfa Weigla 12, 53-114 Wrocław, Poland; 4 Department of Molecular Techniques, Faculty of Medicine, Wroclaw Medical University, ul. M. Curie-Skłodowskiej 52, 50-369 Wrocław, Poland

**Keywords:** SARS-CoV-2, COVID-19, real-time polymerase chain reaction, LAMP assay

## Abstract

Since the 2019 novel coronavirus outbreak began in Wuhan, China, diagnostic methods in the field of molecular biology have been developing faster than ever under the vigilant eye of world’s research community. Unfortunately, the medical community was not prepared for testing such large volumes or ranges of biological materials, whether blood samples for antibody immunological testing, or salivary/swab samples for real-time PCR. For this reason, many medical diagnostic laboratories have made the switch to working in the field of molecular biology, and research undertaken to speed up the flow of samples through laboratory. The aim of this narrative review is to evaluate the current literature on laboratory techniques for the diagnosis of SARS-CoV-2 infection available on pubmed.gov, Google Scholar, and according to the writers’ knowledge and experience of the laboratory medicine. It assesses the available information in the field of molecular biology by comparing real-time PCR, LAMP technique, RNA sequencing, and immunological diagnostics, and examines the newest techniques along with their limitations for use in SARS-CoV-2 diagnostics.

## 1. Introduction

When in 2019 a novel virus was uncovered in association with cases of severe pneumonia in Wuhan, China [1,2,3], few would have imagined that by the beginning of 2021 the World Health Organization (WHO, Geneva, Switzerland) would report 123,074,318 confirmed cases of COVID-19 globally, including 2,441,901 deaths [4]. At the time of this manuscript, Italy, the first European country reportedly affected by severe acute respiratory syndrome coronavirus 2 (SARS-CoV-2), reached 3,376,376 cases, with 30,521,774 confirmed in the United States of America [4]. COVID-19 disease is without a doubt a global threat, which caught most countries unprepared for the urgent need for rapid state-of-the-art diagnostic testing [5].

**Figure 1:**
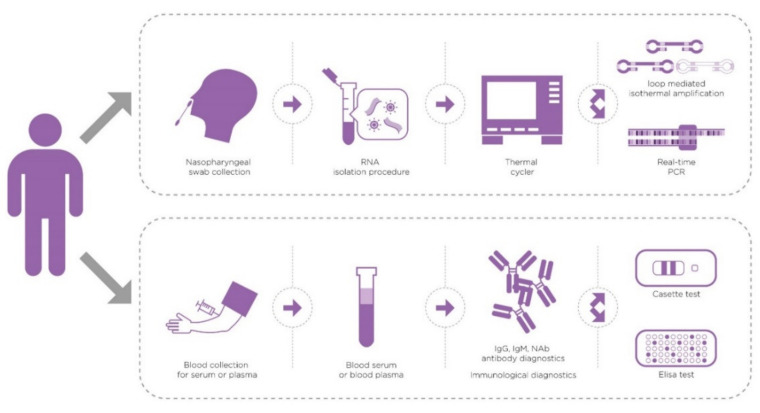
Flow chart of the possible diagnostic processes for SARS-CoV-2.

Despite all the predictions for the longevity of the COVID-19 pandemic and predictions about its future path [6], it is now clear that proper understanding of laboratory medicine should form a cornerstone in safeguarding the future of global health. Regardless of the outcome of the COVID-19 restrictions, lessons need to be learnt about molecular biology techniques, immunological diagnostics, and other laboratory medicine tests used [6,7]. The aim of this review is to evaluate the current literature available on pubmed.gov and Google Scholar on laboratory techniques for the diagnosis of SARS-CoV-2 infection. Additional research through the websites of the World Health Organization, Centers for Disease Control and Prevention, and Food and Drug Administration is provided. Authors compare and examine the limitations of real-time RT-PCR, and RT-LAMP, present the outcomes of antibody/antigen diagnostics, and examine the newest techniques in SARS-CoV-2 diagnostics (Figure 1). This review is designed to be narrative, for the evaluation of current laboratory medicine tests, utilising the available literature, alongside the writers’ knowledge and experience of laboratory medicine.

## 2. Molecular Biology Techniques Targeting SARS-CoV-2 Nucleic Acids

### 2.1. Laboratory Performance of Molecular Tests

Since the worldwide outbreak of COVID-19, there has been a struggle to access sufficient diagnostic resources, including equipment and molecular biology reagents [8,9,10]. This struggle primarily emanates from the initial direction for most of laboratories to use a single diagnostic type, the reverse transcriptase real-time PCR (rRT-PCR) since this method is considered the gold standard for patient diagnostics [5,9,11,12]. However, there have been some attempts to diagnose COVID-19 with other predicting tools [13]; the WHO, in their diagnostic guidelines, have designated molecular biology tests such as the rRT-PCR or RT-LAMP (reverse transcription LAMP) as the NAAT (nucleic acid amplification test), and consider them to be appropriate for SARS-CoV-2 diagnostic purposes [14]. The search for more accurate, less expensive, and faster techniques for the molecular diagnostics of the virus is underway by many scientists worldwide. However, as with all laboratory procedures, there is a need for the standardization of the tests developed, and to this aim the WHO has provided a guidance book for laboratories developing diagnostics for SARS-CoV-2 [15]. With the progression of the pandemics, more patients are seen with high cycle threshold values in the real-time PCR technique. In the case of LAMP, the time needed to reach the level of fluorescence above the cut-off plays the same role as in real-time PCR. For this reason, all the false-positive results need to follow a microbiological scheme of testing, and in the case of uncertainty, the testing needs to be rescheduled.

### 2.2. Preanalytical Errors in SARS-CoV-2 Diagnostics

#### 2.2.1. Patient and Sample Collection-Related Influences on the SARS-CoV-2 Diagnostics

The effectiveness of NAAT techniques is not solely dependent on their specificity and sensitivity, but also on the potential preanalytical errors during the swab collection which could affect the accuracy of the final result [16,17,18]. False-positive results are likely to be due to the effect of over-specific methods, or bad threshold settings, whilst false-negative results are also dependent on the technique of the swab collection and preanalytical sample handling [16]. Proper nasopharyngeal swab operation should result in the collection of an adequate number of cells from the nasopharyngeal tract where the viral load is the highest due to the concentration of ACE receptors [19]. Some problems encountered during the swab collection cannot be avoided such as patient’s movements, the use of nasal sprays by the patient before the sampling, cigarette smoking, and variations in collection from the operator’s site. The potential preanalytical errors can be illustrated in the testing of patients in the healthcare setting. In accordance with the standard sampling and microbiological assay protocol [16,20], instruction should be given to:

- Patients on how to prepare for the swab collection procedure;
- Healthcare personnel on the proper nasopharyngeal collection procedure, management prior the RNA extraction and short-term biobanking before testing [21].

The percentage of false-negative tests from swabs can be further reduced by recommending the use of more than two NAATs prior to patient discharge, and/or that the region of swabbing should be changed to ensure the result is sensitive enough [22]. By following these procedures, the release of patients still positive for SARS-CoV-2 could be notably reduced [16,20].

As even more patients are discharged with the progression of the pandemic, medicine increasingly relies on the molecular diagnostics to provide a reliable source of results, assuring that the patient returns a true-negative upon discharge from the hospital and represents no infectious threat to others [16]. In order to avoid complicated and unpleasant swab collection procedures, to reduce personnel exposure to the virus, and to speed up the sample collection, manufacturers have developed NAAT assays in which a saliva sample can be used for the detection of SARS-CoV-2, which have been proven to provide accurate results [23,24]. 

#### 2.2.2. Timing and Diversity of Diagnostic Biomaterial for the SARS-CoV-2 Testing

With the progression of the pandemic and for better understanding of the disease itself, some materials other than a nasopharyngeal swab can be evaluated for the purpose of SARS-CoV-2 diagnostics. Of much scientific interest are clinical specimens from the lower respiratory tract such as sputum, bronchoalveolar lavage fluid (BAL/BLF), fibrobronchoscopy brush biopsy (FBB) which are used for their clinical relevance evaluation [18,25]. The viral load from the upper respiratory tract (URT) was evaluated to be lower than that from the lower respiratory tract (LRT) [18,25]; however, the URT is considered to be safer for the material probing process. As the detection profile of SARS-CoV-2 in different biomaterials with the use of the rRT-PCR is not clearly established [25], the differences between samples in regards of their origin have to be taken into consideration, when viral diagnostic procedure is to be established. 

Reports about BAL diagnostics from symptomatic patients provided rRT-PCR results, targeting the ORF1ab gene, show a higher positive rate compared to the FBB [26]. However, the specimens of choice for SARS-CoV-2 testing are nasopharyngeal and oropharyngeal swabs. As provided by Gualano et al., BAL specimens should be collected only when it is clinically required [27].

Sputum is a specimen excreted from the LRT, which might be rich in host cells and microorganisms causing the pulmonary/bronchial disease. Its structure is different, and the molecular diagnostics of this biomaterial provide different outcomes than saliva [18]. Sputum was evaluated to be the second best material for SARS-CoV-2 diagnostics by Wang et al. (after BAL), with a 72% conformity in positive rates [26]. Additionally, the SARS-CoV-2 viral load in this biomaterial was diagnosable longer than in nasopharyngeal swabs [1]. However, the induction of sputum is not recommended when diagnosing SARS-CoV-2 [27], and the protocols diminishing the viscosity of this biomaterial need to be followed. This limitation can interfere with its use in coronavirus diagnostics, especially when automatic RNA-isolation are performed.

There are some reports providing that, in the case of negative sampling from URT, SARS-CoV-2 was detected in other biomaterials such as the stool or blood of infected patients [18,25]. One of those biomaterials that might represent a challenge for the molecular diagnostics of RNA is the stool sample. However, its diagnostics could provide an insight into gastrointestinal tract manifestations, and about the eventual faecal–oral transmission [18,26], or the eventual COVID-19 disease progression [28,29]. What needs to be underlined is that the clinical relevance of those samples with the evaluation of their virulence could only be provided by thorough laboratory diagnostics, evaluating whether only the RNA of the inactivated virus was detected, or whether virus cells were present. Additionally, each time any biomaterial is prepared, a thorough preparation of the sample for analysis should be established in the diagnostic laboratory to exclude, e.g., the possibility of test inhibition.

### 2.3. Viral RNA Isolation and Direct PCR Technique

With developments in molecular biology, many techniques can now be carried out in an automated instead of manual way, depending on the resources of the laboratory. There are many benefits to this approach, including speed, accuracy, and a reduction in the exposure of laboratory personnel to the virus. On the market, there are many RNA extraction apparatuses that could be implemented in SARS-CoV-2 diagnostics, with some examples such as the QIAcube Connect (Qiagen, Hilden, Germany), EZ1 Advanced XL (Qiagen) [30], NucliSENS easyMAG (bioMerieux, Marcy-l’Étoile, France) [10], EMAG (bioMerieux) [31], and also those adapted to higher biosafety levels in instruments such as the MagNA Pure LC (Roche Diagnostics, Basel, Switzerland) [32].

To shorten the time needed for the diagnostics to be completed, methods avoiding viral nucleic acid extraction have been tested in direct NAAT approaches [10,25]. Different laboratory schemes have been assessed such as diluting the samples in different mediums (e.g., Hanks’ Balanced Salts, universal transport medium, water for molecular biology, or saline buffers), heat-processed methods [10,25], and the use of Chelex in combination with incubation at temperatures circa 70 °C and centrifugation [10]. These wide-ranging attempts at developing alternative methods in the extraction/direct processing protocols not only lower the time for the result to be obtained, but also help to address the acute shortage of standard commercial viral isolation kits.

### 2.4. Target Genes for SARS-CoV-2 Diagnostics

The single stranded RNA genome of SARS-CoV-2 is built up of circa 30,000 nucleotides [33]. Genes in its sequences encode for 29 different proteins, including those responsible for structural elements of the virus such as the envelope (protein E), spike (protein S), membrane (protein M) and the nucleocapsid (protein N) [33]. The gene regions most targeted by NAAT SARS-CoV-2 diagnostics include the RNA-dependent RNA polymerase—RdRP (the protein product of the cleavage of the polyproteins 1a and 1ab from the conserved replicase domains ORF1a and ORF1b [18,34])—and the previously described structural genes (E, S, N, M) [14,18]. Optimal NAAT diagnostics consist of assays based on two or more independent targets of the SARS-CoV-2 genome in order to provide reliable specificity regardless of the risk viral genome mutation [14]. In early 2021, with new variants of SARS-CoV-2 spreading around the globe, this technique based on multiple target genes has become increasingly crucial [35]. However, it is worth noting that as described by the WHO, in areas with a widespread transmission of SARS-CoV-2, a single target assay might be adopted if strategies for monitoring mutations in the viral genome are also followed [14].

### 2.5. Control Material and Availability of Reference Panels for Assay Confirmation

Each molecular biology NAAT-based technique, especially in the early stages of development, requires reference material for the qualitative detection of nucleic acid of the target pathogen, and more crucially to utilise an additional quantified control in order to establish the limit of detection [30,36]. At the beginning of the pandemic, validation data were obtained from artificial specimens derived from SARS-CoV-2 RNA. Later, the US Food and Drug Administration recommended in their Policy for Coronavirus Disease-2019 Tests that developers could also use patient specimens to validate their tests [37]. However, the FDA, in collaboration with the Centre for Devices and Radiological Health (CDRH) and the Centre for Biologics Evaluation and Research (CBER), has now gone on to develop a SARS-CoV-2 reference panel to fulfil this role. The reference panel allows a comparison of the cross-reactivity and sensitivity of nucleic acid-based SARS-CoV-2 tests by utilising known and blinded viral material [38]. The blinding is provided by the delivery of four vials (named T2, T3, T4, T5) that contain concentrations of the viral RNA known only to the FDA, and one vial (named T1) containing a strain SARS-CoV-2 (provided with the number 2019-nCoV/USA-WA1/2020) at ~1.8 × 10^8^ RNA NAAT detectable units/mL (NDU/mL) [38]. In such a way, the assay under development can be assessed using the control material, and the sensitivity verified using the blinded samples [38]. Unfortunately, the utility of the reference panel does not extend to the stage of assay development which deals with viral mutations and, therefore, it is even more important that analysis of SARS-CoV-2 variants is also performed [38].

In early 2021, the WHO’s International Laboratory for Biological Standards provided the first International RNA Standard for SARS-CoV-2 diagnostics [39]. The England/02/2020 isolate is a lyophilised, heat-acid-inactivated SARS-CoV-2 virus containing a background sequence of human genomic DNA at a concentration of 1 × 10^5^ copies/mL. The reason for the use of this one standard worldwide, is the ability to access the consensus sequences of the RNA from GenBank (reference number MW059036). This new standard is not only lower in cost, but also solves many problems associated with the use of standards such as the viral concentration being provided in different units and a variety of base structures—cDNA, synthetic RNA, viral genome fragments. However, where viral diversity is being evaluated—and for other research purposes—, there are different strains available that are deposited by the Centers for Disease Control and Prevention and obtained via BEI Resources, NIAID, NIH. Available standards are covered in Table 1.

### 2.6. RT-PCR and RT-LAMP Diagnostics for SARS-CoV-2

The current clinical laboratory analysis of SARS-CoV-2 nucleic acids is primarily performed using rRT-PCR, RT-LAMP and, in some cases, next generation sequencing (NGS). Although RNA sequencing is used in a wide range of scientific research, in the pragmatic clinical world, it is strictly targeted at identifying mutations in the viral genome [33,49] and in analysing the spread of the virus within populations by phylogenetic analysis of viral isolates [1,2,50]. The limited use of sequencing is likely related to the high cost and low availability of the equipment, and the relative rarity of laboratory personnel trained in this technique. As the pandemic progresses, there is an increasing need and demand for rapid and sensitive diagnostics, not only to facilitate clinical care with point of care testing (POCT), but also for a wide range of non-clinical roles such as before flights or at sporting events [51,52].

rRT-PCR is the current gold standard for SARS-CoV-2 detection. The well-established, off-the-shelf diagnostic kits include the reverse transcription and amplification enzymes, sets of primers and probes for the amplification of specific viral genome regions, and the authorized reagents for negative, positive, and internal controls. The controls undertaken ensure the quality of the diagnostic process and are conducted in the same manner at the same time as the clinical patient samples. All controls evaluated during the same time frame must provide the required result to validate the outcome of a clinical patient sample (e.g., negative for no template control—NTC—, and positive for the control containing specific viral regions) [7].

Loop-mediated isothermal amplification of nucleic acids (LAMP) was created by Japanese researchers in 2000. It is a fast and efficient method of DNA analysis performed at a single stable temperature and is reported to be able to detect just six copies of the target sequence within a sample [53]. The technique utilizes two to three different pairs of primers to sequentially amplify the target sequence, leading to an accumulation of 109 copies of target DNA in under an hour. The final products are stem-loop DNAs consisting of several inverted repeats of the target, forming a structural amplicon with a cauliflower-like conformation [53]. Reverse transcriptase LAMP (RT-LAMP) is a diagnostic alternative to real-time PCR, and it is able to be used in portable appliances, thus, increasing the speed and ease of SARS-CoV-2 diagnosis [51,54]. Unlike the rRT-PCR, RT-LAMP does not require variations in the thermal profiles and, hence, thermal cyclers, and is more resilient to the sample associated inhibitors which can interfere with the efficiency of rRT-PCR [51,55,56]. The advantages of RT-LAMP mean that onsite diagnostic testing can be performed directly even from saliva samples in just 1–2 h with the time of genetic material isolation included [48].

The FDA provides a list of 195 developers who have utilised the FDA SARS-CoV-2 reference panel, with the majority used in the development of rRT-PCR techniques, and only the minority representing the use of RT-LAMP [38]. Despite the benefits, there still remains some hesitancy in shifting from rRT-PCR to RT-LAMP-based technology as the gold standard in diagnostics. However, it cannot be ignored that RT-LAMP provides some distinct advantages over rRT-PCR, including the improved laboratory workflow and the reduced false-negative results that might be present in rRT-PCR [16] (see Table 2).

Any evaluation of RT-LAMP technology should highlight the importance of correct primer design and correct optimalisation processes. Unlike the construction of primers for PCR diagnostics, the alignment and specificity of the LAMP primers with the conserved regions of the viral genomes are not the only parameters assuring success [57]. Due to the randomness of the results obtained even with the use of specific and correct (in silico) LAMP primers, multiple changes in the primers’ design have to be performed before their use in patients’ diagnostics [57,58,59]. Research conducted to date show several attempts at primer design must be performed before the ideal primer set is chosen for SARS-CoV-2 RT-LAMP diagnostics [60]. In silico alignment alone is not enough, and the laboratory evaluation of prospective primers has to be performed during the optimalisation process [57,59]. The most commonly used tool for the preparation of the RT-LAMP primers is PrimerExplorer, a free program available online, developed by Eiken Chemical Co., Ltd. (Tokyo, Japan) [61], or alternatively the Oligo 7 program (Molecular Biology Insights, Inc., Colorado Springs, CO, USA) [62].

In regard to the RT-LAMP test’s specificity, the limit of detection (LOD) of SARS-CoV-2 viral copies varies for each of the primers designed. Lu et al. constructed primers with an LOD established at a concentration of 118.6 copies per 25 µL reaction [57]. Whilst Huang et al. provided the possibility of detecting two copies of target RNA per 25 µL reaction, the concentration of the RNA, however, was measured via a Nanodrop apparatus, not a digital PCR [60]. To evaluate the RT-LAMP tests, the authors utilized clinical samples to provide a comparison between RT-LAMP and rRT-PCR. Lu et al. evaluated 56 clinical samples from two groups of patients: (i) COVID-19 suspected patients and (ii) control populations. As stated by the authors, the concordance rate between rRT-PCR and RT-LAMP assays was evaluated at 92.9% [57]. Huang et al. undertook the evaluation of RT-LAMP in comparison to rRT-PCR by testing 16 clinical samples (half of which were positive and the other half negative); however, the statistical analysis of concordance between tests was not provided [60]. What has to be kept in mind, is that the statements regarding the clinical utility of the tests are highly likely to be overestimated if the clinical samples were not laboratory evaluated. This can be seen in several studies, including Wang et al. who showed an LOD of six copies of SARS-CoV-2 [63], the laboratory manual presented by Park et al. reporting 100 copies per reaction [59], and lastly, Annamalai et al. detecting 100 copies for the N gene and 1000 copies for the ORF gene in a 25 µL reaction (using colorimetric LAMP detection) [58]. The clinical sample evaluation and specific parameters of RT-LAMP in comparison to real-time PCR, for all beforementioned research, are presented in Table 3.

## 3. Serological Tests

As molecular biology laboratories struggled with the overwhelming number of RT-PCR tests for SARS-CoV-2 diagnostics, a range of new assays to facilitate clinical diagnosis were introduced to the laboratory field. Assays based on immunochemical reactions can be divided into those that detect a component of the invading organism (antigen), or those that detect the specific antibody response to the infection, both of which are discussed below.

### 3.1. Detecting Antigens

As described by some research, and as stated by the clinical evaluation of the tests used, antigen tests show a steady, regular detection decline—with a diminishing viral load their sensitivity is evaluated to be lower than rRT-PCR or RT-LAMP techniques [22,56]. However, as the antigen methods have played some role in patient diagnostics since the third and fourth quarter of 2020, the methodologies utilised are evaluated here.

In SARS-CoV-2 virus, there are four primary proteins that can be used for the detection of an active infection:

- N-protein is a nucleocapsid protein which plays a crucial role in virion assembling;
- E-protein is a structural protein
- M-protein is a matrix protein located in the inner layer of the virus;
- S-protein, called the spike, is specific for the coronavirus structure [64,65,66].

SARS-CoV-2 antigens can be detected with the use of immunochromatography techniques. Commercially produced antibodies, specific for viral antigens, are coated on a nitrocellulose membrane within the POCT, and with the application of a sample material the gold-labelled antibody can then make a characteristic complex with the viral antigen which migrates along the membrane. If virus antigens are present, they show up as a result visible to the bare eye. The sensitivity of the antigen test can vary from 34 to 80%, with a specificity of 90.2% to 100% depending on the commercial test used [67]. Rapid antigen tests do not require complicated machinery and, thus, are attractive candidates for POCT [68].

### 3.2. Types of SARS-CoV-2 Laboratory Diagnostics Utilising Antibodies

According to the latest FDA guidance, antibody tests should not be used in the diagnosis of SARS-CoV-2 infection. This guidance concerns tests for all antibody types that are indicative of infection (e.g., IgM, IgG, IgA) [69]. The reasons for caution are three-fold: Firstly, because of the longer time frame taken for the development of an antibody response and for it to reach detectable levels. The serological window for the IgM antibodies is between 6 and 10 days post infection, with a low concentration within the first few days. If used for diagnostic purposes, this would cause an unacceptable delay for the identification of infection, and a significant risk of spread [7]. Secondly, whilst almost all patients raise an immune response of some kind to the infection, not all will create antibodies. Thirdly, until the beginning of 2021, there was no antibody standard available for the anti-SARS-CoV-2 antibody tests, making it difficult to produce the necessary uniformity and standardization [70].

Currently, the most high-profile antibody utilising tests are the lateral flow immunochromatography (LFIA), popularly known as ‘lateral flow’. They are based on nitrocellulose membranes coated with gold nanoparticle-labelled human antibodies which react with the virus antigens based on the plate. As a result of capillary action, the complex migrates through the membrane and forms a visual vertical line that can be easily read by the operator and does not require sophisticated laboratory skills for its evaluation. Sensitivity and specificity of this kind of test varies between 49.3% and 79.3% and from 96.6% to 99.7%, respectively [71,72]. According to the WHO report from 23 September 2020, only one test from SD Biosensor was approved in emergency use for in vitro diagnostics detecting SARS-CoV-2. Specificity of this test was 99.2% and 95.5%, and assay preparation and evaluation lasts 15–30 min. This technology is, then, available to detect the presence of not only viral antigens, but also, by utilising labelled capture antigens, they can detect an IgG and IgM native antibody response to infection.

Chemiluminescence assays (CLIA) are used in the detection of antibodies for SARS-CoV-2 and are performed using a luminophore marker. The Shenzhen YHLO Biotech kit contains two antigens of SARS-CoV-2 coated on magnetic beads. A specific antibody load is calculated based on the relative light units (RLU) measured by the chemiluminescence analyser. In this particular case, the sensitivity of the test was estimated by the producer as 73.3% (IgM) and 76.7% (IgG), and specificity as 92.2% (IgM) and 100% (IgG) [73].

The enzyme-linked immunosorbent assay (ELISA) uses multi-well plates coated with SARS-CoV-2 antigens (for example S1 protein in the test by EUROIMMUN), that bind anti-SARS-CoV-2 antibodies present in serum/plasma. Although the laboratory workflow is a few hours, it does allow the measurement of several different antigens per sample, in numerous samples at the same time with a partially quantitative/qualitative analysis [74,75].

### 3.3. Therapeutic Neutralizing Antibodies (NAbs) against SARS-CoV-2 Testing

In addition, to the previously discussed IgM and IgG antibodies, there is also a need to develop and diagnose the therapeutic neutralizing antibodies (NAbs) against SARS-CoV-2 [76]. NAbs are monoclonal antibodies that are involved in the process of blocking the virus that can be passively transferred into the patient to potentially prevent viral infection, or to treat the disease [77]. The role of these antibodies in blocking the viral infection shows a promising approach in the treatment of viral respiratory infections due to COVID-19 [76]. As described by S. Zost et al., it is possible that human NAbs could be used for the prophylaxis, post-exposure, or treatment of COVID-19. However, the trials of this treatment are still ongoing and it is not yet clear how such treatments can influence the disease’s outcome [78]. In the literature, the most widely discussed antibodies are the SARS-CoV-2 spike (S) protein-targeting monoclonal antibodies (mAbs), known to have a potent neutralizing activity [78,79]. However, Zost et al. also identified several mAbs targeting the S glycoprotein, that exhibited potent neutralizing activity and fully blocked the viral receptor-binding domain (SRBD) from interacting with the human ACE2 receptor. It is suggested that the mAbs named COV2-2196 and COV2-2130 if analysed might provide virus neutralization, leading to the authors concluding that by using a cocktail, the dose of each mAb can be reduced to achieve the same potency of virus neutralization in vitro [78].

### 3.4. Limitations of Immunological Tests in Evaluation of SARS-CoV-2

Although there is now a widespread use of tests for the detection of the production of antibodies in patients infected with SARS-CoV-2, there are several limitations that make their use in SARS-CoV-2 detection problematic. Firstly, the strength of the adaptive immune response is highly dependent on factors such as age, severity of illness, diet and comorbidities [80,81]. Secondly, there are a number of sensitive points during sample preparation and workflows that can negatively affect the specificity and sensitivity [81,82], requiring qualified personnel to perform and provide a correct interpretation of the results.

What is more, the delay in antibody production in comparison to the ability to detect viral RNA means the window of use for immunological assays is later than for NAAT [80]. After primary infection by the virus, IgM antibodies will be produced first by the patient’s immune system but will not persist for long. This is followed by the production of IgG antibodies which remain for a longer period of time. Unfortunately, as SARS-CoV-2 specific antibodies’ kinetics are still under investigation, a combined IgG and IgM test is needed to correctly and retrospectively diagnose SARS-CoV-2 infection.

## 4. Conclusions

Achievements of laboratory diagnostics in the field of SARS-CoV-2 provide thorough and accurate results when properly optimized (see Table 4). The molecular tests for direct viral detection, such as rRT-PCR, RT-LAMP, could be directly compared when the same RNA standard is used for the reaction optimalisation. Antigen tests should be investigated with care when asymptomatic patients are diagnosed due to a higher limit of detection when compared to NAAT assays. Serological diagnostics, such as semi-quantitative antibody testing, provide an insight on the patient’s disease process. All of these methods have different modes of patients’ preparation for the testing, and different preanalytical parameters should be taken into consideration.

## Figures and Tables

**Table 1:** Genome isolates, and gene fragments of SARS-CoV-2 available from the American Type Culture Collection (ATCC).

Name of the Isolate		Type of the Isolate	Additional Information	Biosafety Level	References
**Strain name**	2019-nCoV/USA-WA1/2020	Whole genome	(GenBank) number: MN985325.1	BSL 2	[40]
**Commercial name**	Genomic RNA from severe acute respiratory syndrome-related coronavirus 2 (ATCC^®^ VR-1986D™)				
**Strain name:**	Hong Kong/VM20001061/2020	Whole genome	A total of six passages with single nucleotide polymorphisms and one 27-base pair deletion in the ORF6 region.	BSL 2	[41]
**Commercial name**	Genomic RNA from severe acute respiratory syndrome-related coronavirus 2 (ATCC^®^ VR-1991D™)				
**Strain name:**	2019-nCoV/Italy-INMI1	Whole genome	-	BSL 2	[42]
**Commercial name**	Genomic RNA from severe acute respiratory syndrome-related coronavirus 2 (ATCC^®^ VR-1992D™)				
**Strain name:**	Germany/BavPat1/2020	Whole genome	Presence of the D614G mutation in this isolate	BSL 2	[43]
**Commercial name**	Genomic RNA from severe acute respiratory syndrome-related coronavirus 2 (ATCC^®^ VR-1994D™)				
**Strain name:**	2019-nCoV/USA-WA1/2020	Whole genome	Heat-inactivated, clarified cell lysate and supernatant from Vero E6 cells infected with SARS-CoV-2	BSL 1	[44]
**Commercial name**	Heat-inactivated SARS-CoV-2 (ATCC^®^ VR-1986HK™)				
**Name of the Genome Fragment**		**Type of the Fragment**	**Concentration Range**	**Biosafety Level**	**References**
**Quantitative Synthetic SARS-CoV-2 RNA: ORF, E, N**		ORF 1ab, Envelope, and Nucleocapsid regions	1 × 10^5^ to 1 × 10^6^ copies/µL	BSL 1	[45]
**Quantitative Synthetic SARS-CoV-2 RNA: Spike 5** **′**		5′ Glycoprotein (Spike) region	1 × 10^5^ to 1 × 10^6^ copies/µL	BSL 1	[46]
**Quantitative Synthetic SARS-CoV-2 RNA: Spike 3** **′**		3′ Glycoprotein (Spike) region	Specification range: 1 × 10^5^ to 1 × 10^6^ copies/µL	BSL 1	[47]
**Quantitative Synthetic SARS-CoV-2 RNA: nsp9, nsp12 (RdRp)**		Fragments from the nsp9 and nsp12 (RdRp) regions	Specification range: 1 × 10^5^ to 1 × 10^6^ copies/µL	BSL 1	[48]

**Table 2:** Comparison of the RT-LAMP and rRT-PCR techniques.

Molecular Technique Involved	Time for the Amplification Process	Number of Primers Involved	Laboratory Equipment Required	Temperature	Reverse Transcriptase Reaction
RT-LAMP	≤15 min	A total of 4–6 for each target gene	PCR thermal cycler, or heating block	Stable, 60–70 °C	Within the cDNA amplification
rRT-PCR	≤90 min	A total of 2 for each target gene	PCR thermal cycler	Depending on cycle varies from 50 to 70 °C	In the separate temperature process

**Table 3:** Parameters of detection for the RT-LAMP reaction and its clinical evaluation.

Reference	Per Reaction Limit of Detection (LOD) (in Copies Per Reaction, See Volume of Reaction)	Reaction Volume	Clinical Human Samples Evaluation	
			Number of Samples	Concordance between RT-LAMP and RT-PCR
[57]	118.6	25 µL	56	92.9%
[63]	6	50 µL	NP *	
[59]	100	15 µL	NP *	
[60]	2	25 µL	16	100%
[58]	>10	25 µL	NP *	

* NP: not performed.

**Table 4:** Short description of mostly used techniques in the SARS CoV-2 diagnostics.

**SARS CoV-2 Diagnostics**	**Type of the Technique (Acronym)**	**Full Name of the Technique**	**Short Description of the Procedure**	**Scope of the Diagnostics**	**Type of Diagnostics: Laboratory Procedure or Point of Care (POCT)**	**Possible Biomaterial to Be Used**	**Appropriate Timing for the Diagnostics**
	rRT-PCR [30]	Reverse transcription real-time polymerase chain reaction	Two primer-based technique directed to specific SARS-CoV-2 RNA sequence	Gold standard of the SARS-CoV-2 diagnostics	Laboratory procedure	Saliva, nasopharyngeal swab, pharyngeal swab	A total of 1–3 days from the onset of symptoms [83]
	NGS [50]	Next-generation sequencing	Simultaneous analysis of different sequences revealing specific RNA sequences	Whole genome and gene fragments sequencing, e.g., in the case of the new infections of variant diagnostics (surveillance studies)	Laboratory procedure	Saliva, nasopharyngeal swab, pharyngeal swab	A total of 1–3 days from the onset of symptoms [83]
	RT-LAMP [55]	Reverse transcription Loop-Mediated Isothermal Amplification	Eight primer-based techniques directed to specific SARS-CoV-2 RNA sequence	Fast fluorescent or probe-based specific amplification	Laboratory procedure and POCT	Saliva, nasopharyngeal swab, pharyngeal swab	A total of 1–3 days from the onset of symptoms [83]
	CRISPR Cas9 [82]	Clustered regularly interspaced short palindromic repeats	Probing of the amplified specific fragments of the virus after the activity of proteinases with sgRNA complex	Patient’s diagnostics and treatment research	Laboratory procedure and POCT	Saliva, nasopharyngeal swab, pharyngeal swab	* NA
	Ag-tests [22]	Antigen tests	Tests providing results for the antigen’s characteristic for the SARS CoV-2 diagnostics	Symptomatic patients, and fast-track diagnostics	Laboratory procedure and POCT	Nasopharyngeal swab, saliva	A total of 3–4, or up to 7 days from the symptoms onset [83]
**Antibody during the process of the SARS CoV-2 infection**	Nabs [76]	Therapeutic neutralizing antibodies	Human monoclonal antibodies targeting specific protein of the viral capsid	Important during tests of the immunity after the vaccination	Laboratory procedure	Serum and/or plasma	Seroconversion observed after 5.5 days [84]
	Ab-tests [73]	IgG, IgM, IgA, etc., antibody diagnostics	Analysis of antibodies produced by healed, infected, vaccinated subjects.	When appropriately specific, tracking primary and secondary immunological response in the population for SARS-CoV-2	Laboratory procedure and POCT	Serum and/or plasma	Seroconversion observed after 7 to 14 days for IgG and IgM [83]

* NA: data not available.

## Data Availability

No additional data available.

## References

[1] Caly L., Druce J., Roberts J., Bond K., Tran T., Kostecki R., Yoga Y., Naughton W., Taiaroa G., Seemann T. (2020). Isolation and rapid sharing of the 2019 novel coronavirus (SARS-CoV-2) from the first patient diagnosed with COVID-19 in Australia. Med. J. Aust..

[2] Park W.B., Kwon N.J., Choi S.J., Kang C.K., Choe P.G., Kim J.Y., Yun J., Lee G.W., Seong M.W., Kim N.J. (2020). Virus isolation from the first patient with SARS-CoV-2 in Korea. J. Korean Med. Sci..

[3] Deng S.-Q., Peng H.-J. (2020). Characteristics of and Public Health Responses to the Coronavirus Disease 2019 Outbreak in China. J. Clin. Med..

[4] World Health Organization WHO Coronavirus Disease (COVID-19) Dashboard. https://covid19.who.int/?gclid=CjwKCAjwnK36BRBVEiwAsMT8WJ3y00_BUzvrLsvbl3uthuoTH_Occ45gyEUbpYRyEqAzll3aZB6TYxoCcM0QAvD_BwE.

[5] Younes N., Al-SAdeq D.W., Al-Jighefee H., Younes S., Al-Jamal O., Daas H.I., Yassine H.M., Nasrallah G.K. (2020). Challenges in Laboratory Diagnosis of the Novel Coronavirus SARS-CoV-2. Viruses.

[6] Estrada E. (2020). COVID-19 and SARS-CoV-2. Modeling the present, looking at the future. Phys. Rep..

[7] Yüce M., Filiztekin E., Özkaya K.G. (2021). COVID-19 diagnosis —A review of current methods. Biosens. Bioelectron..

[8] Sullivan P.S., Sailey C., Guest J.L., Guarner J., Kelley C., Siegler A.J., Valentine-Graves M., Gravens L., del Rio C., Sanchez T.H. (2020). Detection of SARS-CoV-2 RNA and Antibodies in Diverse Samples: Protocol to Validate the Sufficiency of Provider-Observed, Home-Collected Blood, Saliva, and Oropharyngeal Samples. JMIR Public Heal. Surveill..

[9] Moreno-Contreras J., Espinoza M.A., Sandoval-Jaime C., Cantú-Cuevas M.A., Barón-Olivares H., Ortiz-Orozco O.D., Muñoz-Rangel A.V., Hernández-de la Cruz M., Eroza-Osorio C.M., Arias C.F. (2020). Saliva sampling and its direct lysis, an excellent option to increase the number of SARS CoV2 diagnostic tests in settings with supply shortages. J. Clin. Microbiol..

[10] Ulloa S., Bravo C., Parra B., Ramirez E., Acevedo A., Fasce R., Fernandez J. (2020). A simple method for SARS-CoV-2 detection by rRT-PCR without the use of a commercial RNA extraction kit. J. Virol. Methods.

[11] Seidu A.A., Hagan J.E., Ameyaw E.K., Ahinkorah B.O., Schack T. (2020). The role of testing in the fight against COVID-19: Current happenings in Africa and the way forward. Int. J. Infect. Dis..

[12] Gopalkrishnan M., Krishna S. (2020). Pooling Samples to Increase SARS-CoV-2 Testing. J. Indian Inst. Sci..

[13] Sambataro G., Giuffrè M., Sambataro D., Palermo A., Vignigni G., Cesareo R., Crimi N., Torrisi S.E., Vancheri C., Malatino L. (2020). The Model for Early COvid-19 Recognition (MECOR) Score: A Proof-of-Concept for a Simple and Low-Cost Tool to Recognize a Possible Viral Etiology in Community-Acquired Pneumonia Patients during COVID-19 Outbreak. Diagnostics.

[14] World Health Organization (2020). WHO Diagnostic Testing for SARS-CoV-2.

[15] WHO (2020). Laboratory biosafety guidance related to coronavirus disease 2019 (COVID-19). Interim. Guid..

[16] Dao T.L., Hoang V.T., Gautret P. (2021). Recurrence of SARS-CoV-2 viral RNA in recovered COVID-19 patients: A narrative review. Eur. J. Clin. Microbiol. Infect. Dis..

[17] Wernike K., Keller M., Conraths F.J., Mettenleiter T.C., Groschup M.H., Beer M. (2020). Pitfalls in SARS-CoV-2 PCR diagnostics. Transbound. Emerg. Dis..

[18] Yan Y., Chang L., Wang L. (2020). Laboratory testing of SARS-CoV, MERS-CoV, and SARS-CoV-2 (2019-nCoV): Current status, challenges, and countermeasures. Rev. Med. Virol..

[19] Xu H., Zhong L., Deng J., Peng J., Dan H., Zeng X., Li T., Chen Q. (2020). High expression of ACE2 receptor of 2019-nCoV on the epithelial cells of oral mucosa. Int. J. Oral Sci..

[20] Zou Y., Wang B.R., Sun L., Xu S., Kong Y.G., Shen L.J., Liang G.T., Chen S.M. (2020). The Issue of Recurrently Positive Patients Who Recovered from COVID-19 According to the Current Discharge Criteria: Investigation of Patients from Multiple Medical Institutions in Wuhan, China. J. Infect. Dis..

[21] Lippi G., Simundic A.M., Plebani M. (2020). Potential preanalytical and analytical vulnerabilities in the laboratory diagnosis of coronavirus disease 2019 (COVID-19). Clin. Chem. Lab. Med..

[22] Hirotsu Y., Maejima M., Shibusawa M., Nagakubo Y., Hosaka K., Amemiya K., Sueki H., Hayakawa M., Mochizuki H., Tsutsui T. (2020). Comparison of automated SARS-CoV-2 antigen test for COVID-19 infection with quantitative RT-PCR using 313 nasopharyngeal swabs, including from seven serially followed patients. Int. J. Infect. Dis..

[23] Azzi L., Maurino V., Baj A., Dani M., d’Aiuto A., Fasano M., Lualdi M., Sessa F., Alberio T. (2021). Diagnostic Salivary Tests for SARS-CoV-2. J. Dent. Res..

[24] Azzi L., Carcano G., Gianfagna F., Grossi P., Gasperina D.D., Genoni A., Fasano M., Sessa F., Tettamanti L., Carinci F. (2020). Saliva is a reliable tool to detect SARS-CoV-2. J. Infect..

[25] Bwire G.M., Majigo M.V., Njiro B.J., Mawazo A. (2021). Detection profile of SARS-CoV-2 using RT-PCR in different types of clinical specimens: A systematic review and meta-analysis. J. Med. Virol..

[26] Wang W., Xu Y., Gao R., Lu R., Han K., Wu G., Tan W. (2020). Detection of SARS-CoV-2 in Different Types of Clinical Specimens. JAMA J. Am. Med. Assoc..

[27] Gualano G., Musso M., Mosti S., Mencarini P., Mastrobattista A., Pareo C., Zaccarelli M., Migliorisi P., Vittozzi P., Zumla A. (2020). Usefulness of bronchoalveolar lavage in the management of patients presenting with lung infiltrates and suspect COVID-19-associated pneumonia: A case report. Int. J. Infect. Dis..

[28] Giuffrè M., Di Bella S., Sambataro G., Zerbato V., Cavallaro M., Occhipinti A.A., Palermo A., Crescenzi A., Monica F., Luzzati R. (2020). COVID-19-Induced thrombosis in patients without gastrointestinal symptoms and elevated fecal calprotectin: Hypothesis regarding mechanism of intestinal damage associated with COVID-19. Trop. Med. Infect. Dis..

[29] Giuffrè M., Bozzato A.M., Di Bella S., Occhipinti A.A., Martingano P., Cavallaro M.F.M., Luzzati R., Monica F., Cova M.A., Crocè L.S. (2020). Spontaneous rectal perforation in a patient with SARS–CoV-2 infection. J. Pers. Med..

[30] Pujadas E., Ibeh N., Hernandez M.M., Waluszko A., Sidorenko T., Flores V., Shiffrin B., Chiu N., Young-Francois A., Nowak M.D. (2020). Comparison of SARS-CoV-2 detection from nasopharyngeal swab samples by the Roche cobas 6800 SARS-CoV-2 test and a laboratory-developed real-time RT-PCR test. J. Med. Virol..

[31] Barza R., Patel P., Sabatini L., Singh K. (2020). Use of a simplified sample processing step without RNA extraction for direct SARS-CoV-2 RT-PCR detection. J. Clin. Virol..

[32] Chiu R.W.K., Jin Y., Chung G.T.Y., Lui W.B., Chan A.T.C., Lim W., Lo Y.M.D. (2006). Automated extraction protocol for quantification of SARS-coronavirus RNA in serum: An evaluation study. BMC Infect. Dis..

[33] Biswas N.K., Majumder P.P. (2012). Analysis of RNA sequences of 3636 SARS-CoV-2 collected from 55 countries reveals selective sweep of one virus type. J. Dent. Educ..

[34] Chan J.F.-W., Yip C.C.-Y., To K.K.-W., Tang T.H.-C., Wong S.C.-Y., Leung K.-H., Fung A.Y.-F., Ng A.C.-K., Zou Z., Tsoi H.-W. (2020). Improved Molecular Diagnosis of COVID-19 by the Novel, Highly Sensitive and Specific COVID-19-RdRp/Hel Real-Time Reverse Transcription-PCR Assay Validated In Vitro and with Clinical Specimens. J. Clin. Microbiol..

[35] Peñarrubia L., Ruiz M., Porco R., Rao S.N., Juanola-Falgarona M., Manissero D., López-Fontanals M., Pareja J. (2020). Multiple assays in a real-time RT-PCR SARS-CoV-2 panel can mitigate the risk of loss of sensitivity by new genomic variants during the COVID-19 outbreak. Int. J. Infect. Dis..

[36] Hirotsu Y., Maejima M., Shibusawa M., Nagakubo Y., Hosaka K., Amemiya K., Sueki H., Hayakawa M., Mochizuki H., Tsutsui T. (2020). Pooling RT-qPCR testing for SARS-CoV-2 in 1000 individuals of healthy and infection-suspected patients. Sci. Rep..

[37] FDA Policy for Diagnostic Tests for COVID-19 during the Public Health Emergency 2020. https://Www.Fda.Gov/Regulatory-Information/Search-Fda-Guidance-Documents/Policy-Coronavirus-Disease-2019-Tests-During-Public-Health-Emergency-Revised.

[38] FDA SARS-CoV-2 Reference Panel Comparative Data on This Page: Development of the FDA SARS-CoV-2 Reference Panel. https://www.fda.gov/medical-devices/coronavirus-covid-19-and-medical-devices/sars-cov-2-reference-panel-comparative-data#development.

[39] WHO (2021). International Standard First WHO International Standard for SARS-CoV-2 RNA.

[40] American Type Culture Collection (2020). Genomic RNA from Severe acute respiratory syndrome-related coronavirus 2 (ATCC® VR-1986D^TM^). https://www.atcc.org/products/vr-1986d.

[41] American Type Culture Collection (2020). Genomic RNA from Severe acute respiratory syndrome related coronavirus 2 (ATCC® VR 1991D^TM^). https://www.atcc.org/products/vr-1991d.

[42] American Type Culture Collection (2020). Genomic RNA from Severe acute respiratory syndrome-related coronavirus 2 (ATCC® VR-1992D^TM^). https://www.atcc.org/products/vr-1992d.

[43] American Type Culture Collection (2020). Genomic RNA from Severe acute respiratory syndrome-related coronavirus 2 (ATCC® VR-1994DTM). https://www.atcc.org/products/vr-1994d.

[44] American Type Culture Collection (2020). Heat-inactivated SARS-CoV-2 (ATCC® VR-1986HK^TM^). https://www.atcc.org/products/vr-1986hk.

[45] American Type Culture Collection (2020). Quantitative Synthetic SARS-CoV-2 RNA: ORF, E, N (ATCC® VR-3276SD^TM^). https://www.atcc.org/products/vr-3276sd.

[46] American Type Culture Collection (2020). Quantitative Synthetic SARS CoV 2 RNA:ORF, E, N. https://www.atcc.org/products/vr-3277sd.

[47] American Type Culture Collection (2020). Quantitative Synthetic SARS?CoV-2 RNA: Spike 3’ (ATCC® VR-3278SD^TM^). https://www.atcc.org/products/vr-3278sd.

[48] American Type Culture Collection (2020). Quantitative Synthetic SARS CoV 2 RNA: nsp9, nsp12 (RdRp) (ATCC ® VR 3279SD ^TM^). Am. Type Cult. Collect..

[49] Stefanelli P., Faggioni G., Lo Presti A., Fiore S., Marchi A., Benedetti E., Fabiani C., Anselmo A., Ciammaruconi A., Fortunato A. (2020). Whole genome and phylogenetic analysis of two SARSCoV-2 strains isolated in Italy in January and February 2020: Additional clues on multiple introductions and further circulation in Europe. Eurosurveillance.

[50] To K.K.-W., Hung I.F.-N., Ip J.D., Chu A.W.-H., Chan W.-M., Tam A.R., Fong C.H.-Y., Yuan S., Tsoi H.-W., Ng A.C.-K. (2020). Coronavirus Disease 2019 (COVID-19) Re-infection by a Phylogenetically Distinct Severe Acute Respiratory Syndrome Coronavirus 2 Strain Confirmed by Whole Genome Sequencing. Clin. Infect. Dis..

[51] Ganguli A., Mostafa A., Berger J., Aydin M.Y., Sun F., Stewart de Ramirez S.A., Valera E., Cunningham B.T., King W.P., Bashir R. (2020). Rapid isothermal amplification and portable detection system for SARS-CoV-2. Proc. Natl. Acad. Sci. USA.

[52] Lamb L.E., Bartolone S.N., Ward E., Chancellor M.B. (2020). Rapid detection of novel coronavirus/Severe Acute Respiratory Syndrome Coronavirus 2 (SARS-CoV-2) by reverse transcription-loop-mediated isothermal amplification. PLoS ONE.

[53] Notomi T., Okayama H., Masubuchi H., Yonekawa T., Watanabe K., Amino N., Hase T. (2000). Loop-mediated isothermal amplification of DNA. Nucleic Acids Res..

[54] Janíková M., Hodosy J., Boor P., Klempa B., Celec P. (2021). Loop-mediated isothermal amplification for the detection of SARS-CoV-2 in saliva. Microb. Biotechnol..

[55] Rödel J., Egerer R., Suleyman A., Sommer-Schmid B., Baier M., Henke A., Edel B., Löffler B. (2020). Use of the variplex^TM^ SARS-CoV-2 RT-LAMP as a rapid molecular assay to complement RT-PCR for COVID-19 diagnosis. J. Clin. Virol..

[56] Nagura-Ikeda M., Imai K., Tabata S., Miyoshi K., Murahara N., Mizuno T. (2020). Clinical Evaluation of Self-Collected Saliva by Quantitative Reverse Transcription-PCR (RT-qPCR), Direct RT-qPCR, Reverse Transcription–Loop-Mediated Isothermal Amplification, and a Rapid Antigen Test to Diagnose COVID-19. J. Clin. Microbiol..

[57] Lu R., Wu X., Wan Z., Li Y., Jin X., Zhang C. (2020). A novel reverse transcription loop-mediated isothermal amplification method for rapid detection of sars-cov-2. Int. J. Mol. Sci..

[58] Annamalai P., Kanta M., Ramu P., Ravi B., Veerapandian K., Srinivasan R. (2020). A simple colorimetric molecular detection of novel coronavirus (COVID-19), an essential diagnostic tool for pandemic screening. medRxiv.

[59] Park G.-S., Ku K., Baek S.-H., Kim S.-J., Kim S.I., Kim B.-T., Maeng J.-S. (2020). Development of Reverse Transcription Loop-Mediated Isothermal Amplification Assays Targeting Severe Acute Respiratory Syndrome Coronavirus 2 (SARS-CoV-2). J. Mol. Diagn..

[60] Huang W.E., Lim B., Hsu C.C., Xiong D., Wu W., Yu Y., Jia H., Wang Y., Zeng Y., Ji M. (2020). RT-LAMP for rapid diagnosis of coronavirus SARS-CoV-2. Microb. Biotechnol..

[61] EIKEN CHEMICAL. https://primerexplorer.jp/e/.

[62] DBA. Oligo, Inc 1267 Vondelpark, C.S. Oligo 7 (Molecular Biology Insights, Inc. Colorado Springs, CO, USA). https://www.oligo.net/index.html.

[63] Wang D. (2020). One-pot Detection of COVID-19 with Real-time Reverse-transcription Loop-mediated Isothermal Amplification (RT-LAMP) Assay and Visual RT-LAMP Assay. bioRxiv.

[64] Wang M.-Y., Zhao R., Gao L.-J., Gao X.-F., Wang D.-P., Cao J.-M. (2020). SARS-CoV-2: Structure, Biology, and Structure-Based Therapeutics Development. Front. Cell. Infect. Microbiol..

[65] Ke Z., Oton J., Qu K., Cortese M., Zila V., McKeane L., Nakane T., Zivanov J., Neufeldt C.J., Cerikan B. (2020). Structures and distributions of SARS-CoV-2 spike proteins on intact virions. Nature.

[66] Gozalbo-Rovira R., Gimenez E., Latorre V., Francés-Gómez C., Albert E., Buesa J., Marina A., Blasco M.L., Signes-Costa J., Rodríguez-Díaz J. (2020). SARS-CoV-2 antibodies, serum inflammatory biomarkers and clinical severity of hospitalized COVID-19 patients. J. Clin. Virol..

[67] Rastawicki W., Rokosz-Chudziak N. (2020). Characteristics and assessment of the usefulness of serological tests in the diagnostic of infections caused by coronavirus SARS-CoV-2 on the basis of available manufacturer’s data and literature review. Przegl. Epidemiol..

[68] European Centre for Disease Prevention and Control (2020). Options for the Use of Rapid Antigen Tests for COVID-19 in the EU/EEA and the UK Key messages.

[69] (2020). In Vitro Diagnostics EUAs. https://www.fda.gov/medical-devices/coronavirus-disease-2019-covid-19-emergency-use-authorizations-medical-devices/vitro-diagnostics-euas.

[70] Mattiuzzo G., Bentley E.M., Hassall M., Routley S. (2020). Establishment of the WHO International Standard and Reference Panel for anti-SARS-CoV-2 antibody.

[71] Lisboa Bastos M., Tavaziva G., Abidi S.K., Campbell J.R., Haraoui L.P., Johnston J.C., Lan Z., Law S., MacLean E., Trajman A. (2020). Diagnostic accuracy of serological tests for covid-19: Systematic review and meta-analysis. BMJ.

[72] Flower B., Brown J.C., Simmons B., Moshe M., Frise R., Penn R., Kugathasan R., Petersen C., Daunt A., Ashby D. (2020). Clinical and laboratory evaluation of SARS-CoV-2 lateral flow assays for use in a national COVID-19 seroprevalence survey. Thorax.

[73] Infantino M., Grossi V., Lari B., Bambi R., Perri A., Manneschi M., Terenzi G., Liotti I., Ciotta G., Taddei C. (2020). Diagnostic accuracy of an automated chemiluminescent immunoassay for anti-SARS-CoV-2 IgM and IgG antibodies: An Italian experience. J. Med. Virol..

[74] Roy V., Fischinger S., Atyeo C., Slein M., Loos C., Balazs A., Luedemann C., Astudillo M.G., Yang D., Wesemann D.R. (2020). SARS-CoV-2-specific ELISA development. J. Immunol. Methods.

[75] Weidner L., Gänsdorfer S., Unterweger S., Weseslindtner L., Drexler C., Farcet M., Witt V., Schistal E., Schlenke P., Kreil T.R. (2020). Quantification of SARS-CoV-2 antibodies with eight commercially available immunoassays. J. Clin. Virol..

[76] Liu L.D., Lian C., Yeap L.-S., Meng F.-L. (2020). The development of neutralizing antibodies against SARS-CoV-2 and their common features. J. Mol. Cell Biol..

[77] Jiang S., Zhang X., Yang Y., Hotez P.J., Du L. (2020). Neutralizing antibodies for the treatment of COVID-19. Nat. Biomed. Eng..

[78] Zost S.J., Gilchuk P., Case J.B., Binshtein E., Chen R.E., Nkolola J.P., Schäfer A., Reidy J.X., Trivette A., Nargi R.S. (2020). Potently neutralizing and protective human antibodies against SARS-CoV-2. Nature.

[79] Chi X., Yan R., Zhang J., Zhang G., Zhang Y., Hao M., Zhang Z., Fan P., Dong Y., Yang Y. (2020). A neutralizing human antibody binds to the N-terminal domain of the Spike protein of SARS-CoV-2. Science.

[80] Xie J., Ding C., Li J., Wang Y., Guo H., Lu Z., Wang J., Zheng C., Jin T., Gao Y. (2020). Characteristics of patients with coronavirus disease (COVID-19) confirmed using an IgM-IgG antibody test. J. Med. Virol..

[81] Mak G.C., Cheng P.K., Lau S.S., Wong K.K., Lau C.S., Lam E.T., Chan R.C., Tsang D.N. (2020). Evaluation of rapid antigen test for detection of SARS-CoV-2 virus. J. Clin. Virol..

[82] Alpdagtas S., Ilhan E., Uysal E., Sengor M., Ustundag C.B., Gunduz O. (2020). Evaluation of current diagnostic methods for COVID-19. APL Bioeng..

[83] Jayamohan H., Lambert C.J., Sant H.J., Jafek A., Patel D., Feng H., Beeman M., Mahmood T., Nze U., Gale B.K. (2021). SARS-CoV-2 pandemic: A review of molecular diagnostic tools including sample collection and commercial response with associated advantages and limitations. Anal. Bioanal. Chem..

[84] Broughton J.P., Deng X., Yu G., Fasching C.L., Servellita V., Singh J., Miao X., Streithorst J.A., Granados A., Sotomayor-Gonzalez A. (2020). CRISPR–Cas12-based detection of SARS-CoV-2. Nat. Biotechnol..

